# Supplementary material for: T cell receptor repertoire as a novel indicator for identification and immune surveillance of patients with severe obstructive sleep apnea
Source: PeerJ. 2023 Apr 7;11:e15009. doi: 10.7717/peerj.15009 (PMC10084822; doi:10.7717/peerj.15009)
Supplement: Supplemental Information 1 [file peerj-11-15009-s001.docx]

|  | Healthy Donors | Non-OSA | Mild-to-Moderate OSA | Severe OSA | P value |
| --- | --- | --- | --- | --- | --- |
| Subjects n | 157 | 23 | 23 | 50 |  |
| Clean Reads k | 74.92(55.79, 95.29)^b,c,d^ | 133.65(118.87, 163.80)^a^ | 100.85(83.33, 190.84)^a^ | 145.13(73.18, 174.57)^a^ | **0.000** |
| D50 % | 12.53(7.98, 17.85)^c,d^ | 14.13(10.25, 17.03) | 16.67(11.56, 23.45)^a^ | 15.98(10.62, 21.93)^a^ | **0.017** |
| Shannon | 8.37(7.70, 8.96)^c,d^ | 8.57(8.08, 8.88) | 8.67(8.22, 9.34)^a^ | 8.73(8.17, 9.11)^a^ | **0.016** |
| Clonotypes k | 12.96(7.66, 15.82)^c,d^ | 14.90(12.42, 16.91) | 15.98(14.68, 19.26)^a^ | 15.94(12.83, 18.71)^a^ | **0.000** |
| UniqueVJ | 450.00(405.00, 472.00)^b,c,d^ | 530.00(511.00, 556.00)^a^ | 553.00(545.00, 561.00)^a^ | 545.00(523.00, 557.25)^a^ | **0.000** |
| Significantly different from: HD(a), Non-OSA(b), Mild-to-mderate OSA(c), Severe OSA(d);Method: nonparametic Kruskal-Wallis test | | | | | |

**Table S1： Diversity and Clonality of Subjects**
