# Supplementary material for: T cell receptor repertoire as a novel indicator for identification and immune surveillance of patients with severe obstructive sleep apnea
Source: PeerJ. 2023 Apr 7;11:e15009. doi: 10.7717/peerj.15009 (PMC10084822; doi:10.7717/peerj.15009)
Supplement: Supplemental Information 2 [file peerj-11-15009-s002.docx]

| **Table S2: Different VJ Junction in Severe OSA Compared With Other Groups** | | | | | |
| --- | --- | --- | --- | --- | --- |
| **vs.Mild-to-Moderate OSA** | | **vs. Non-OSA** | | **vs.HD** | |
| PValue | VJ_allele | PValue | VJ_allele | PValue | VJ_allele |
| 6.41E-06 | V25-1-J2-3 | 0.000122288 | V7-9-J1-3 | 1.93E-26 | V28-J1-6 |
| 1.08E-05 | V9-J2-6 | 0.000221573 | V6-6-J2-3 | 1.93E-26 | V27-J2-1 |
| 1.21E-05 | V19-J1-2 | 0.000232153 | V7-9-J1-4 | 1.93E-26 | V28-J1-2 |
| 1.35E-05 | V11-2-J2-3 | 0.000470302 | V7-9-J2-2 | 1.93E-26 | V27-J1-1 |
| 1.67E-05 | V11-3-J1-1 | 0.000561364 | V10-2-J1-6 | 1.93E-26 | V28-J2-7 |
| 1.91E-05 | V5-6-J2-2 | 0.001345039 | V7-9-J2-4 | 1.93E-26 | V28-J2-5 |
| 3.41E-05 | V11-3-J2-3 | 0.001684403 | V6-1-J2-2 | 1.93E-26 | V27-J2-3 |
| 3.59E-05 | V10-3-J2-5 | 0.0018262 | V6-6-J2-2 | 1.93E-26 | V28-J2-1 |
| 3.78E-05 | V9-J2-3 | 0.001901138 | V7-9-J1-1 | 1.93E-26 | V27-J1-2 |
| 3.98E-05 | V25-1-J2-4 | 0.001978892 | V7-2-J2-7 | 1.93E-26 | V12-4-J1-1 |
| 5.98E-05 | V9-J2-2 | 0.002101006 | V6-6-J1-2 | 1.93E-26 | V12-4-J2-3 |
| 6.95E-05 | V11-3-J1-6 | 0.002413279 | V11-1-J2-1 | 1.93E-26 | V28-J2-2 |
| 6.95E-05 | V18-J1-6 | 0.002559677 | V29-1-J1-4 | 1.93E-26 | V28-J2-3 |
| 7.12E-05 | V25-1-J2-7 | 0.003230097 | V7-4-J1-2 | 1.93E-26 | V28-J1-5 |
| 7.30E-05 | V11-2-J2-2 | 0.00355392 | V6-6-J2-7 | 2.02E-26 | V12-4-J2-7 |
| 8.07E-05 | V19-J2-3 | 0.003691519 | V6-6-J1-5 | 2.05E-26 | V6-1-J1-2 |
| 8.48E-05 | V11-3-J2-7 | 0.003907014 | V14-J1-6 | 2.08E-26 | V27-J1-5 |
| 8.48E-05 | V25-1-J2-5 | 0.004710393 | V6-5-J2-2 | 2.11E-26 | V27-J2-5 |
| 0.000105701 | V10-3-J2-3 | 0.004798445 | V6-6-J2-6 | 2.20E-26 | V27-J1-6 |
| 0.000108309 | V10-3-J2-6 | 0.004979032 | V11-1-J2-3 | 2.30E-26 | V28-J1-1 |
| 0.000110979 | V11-3-J2-1 | 0.005660505 | V14-J2-4 | 2.54E-26 | V27-J2-2 |
| 0.000119363 | V18-J2-5 | 0.006425015 | V20-1-J2-7 | 2.74E-26 | V4-1-J2-2 |
| 0.000122288 | V9-J2-1 | 0.007152928 | V7-9-J2-3 | 2.86E-26 | V4-2-J2-2 |
| 0.000134677 | V25-1-J2-6 | 0.007281198 | V6-1-J2-7 | 3.03E-26 | V4-2-J2-1 |
| 0.000144736 | V11-3-J1-2 | 0.007543949 | V9-J1-3 | 3.71E-26 | V4-1-J2-1 |
| 0.00015183 | V11-1-J2-1 | 0.007678486 | V6-6-J2-1 | 3.93E-26 | V6-1-J1-5 |
| 0.00015183 | V10-3-J2-1 | 0.00781517 | V11-1-J2-7 | 4.28E-26 | V12-4-J1-2 |
| 0.000163087 | V15-J1-4 | 0.008531823 | V19-J2-4 | 4.47E-26 | V12-4-J1-5 |
| 0.000171024 | V25-1-J1-5 | 0.008682013 | V10-2-J2-7 | 5.48E-26 | V28-J2-6 |
| 0.000187998 | V18-J2-7 | 0.008834561 | V7-4-J1-4 | 5.80E-26 | V12-4-J2-1 |
| 0.000216454 | V10-3-J2-7 | 0.008989499 | V19-J1-3 | 6.06E-26 | V4-2-J2-7 |
| 0.000221573 | V11-3-J1-3 | 0.008989499 | V2-J1-3 | 6.14E-26 | V27-J2-7 |
| 0.000232153 | V6-9-J1-5 | 0.009468975 | V5-4-J1-1 | 6.60E-26 | V6-1-J1-3 |
| 0.00023762 | V11-3-J1-4 | 0.010143711 | V5-6-J1-1 | 8.08E-26 | V6-1-J2-3 |
| 0.000248917 | V4-3-J1-2 | 0.010318946 | V6-1-J2-1 | 8.31E-26 | V4-2-J1-5 |
| 0.000260716 | V4-1-J2-2 | 0.010677519 | V7-9-J1-5 | 8.93E-26 | V4-1-J2-7 |
| 0.000260716 | V19-J2-5 | 0.011047128 | V6-6-J2-5 | 1.05E-25 | V6-1-J1-6 |
| 0.000279402 | V12-5-J2-2 | 0.011820609 | V6-5-J2-6 | 1.14E-25 | V4-2-J1-1 |
| 0.000279402 | V19-J2-7 | 0.012021331 | V2-J2-2 | 1.16E-25 | V4-1-J1-5 |
| 0.000292549 | V9-J1-2 | 0.01243186 | V2-J1-5 | 1.30E-25 | V4-3-J1-5 |
| 0.000299337 | V4-3-J1-4 | 0.013738906 | V6-5-J2-5 | 1.30E-25 | V4-1-J2-3 |
| 0.000320599 | V4-2-J1-4 | 0.014436841 | V7-6-J2-4 | 1.45E-25 | V4-1-J1-1 |
| 0.00033555 | V11-2-J2-7 | 0.014919328 | V5-1-J1-2 | 1.88E-25 | V6-1-J2-2 |
| 0.000343268 | V11-2-J2-5 | 0.016188207 | V11-1-J2-2 | 1.91E-25 | V4-1-J1-3 |
| 0.000351151 | V5-6-J1-5 | 0.016188207 | V7-4-J2-1 | 1.91E-25 | V4-2-J2-5 |
| 0.000367429 | V9-J2-7 | 0.016721735 | V7-9-J1-2 | 1.99E-25 | V28-J2-4 |
| 0.000367429 | V12-5-J2-1 | 0.016721735 | V6-9-J2-2 | 2.14E-25 | V4-1-J1-2 |
| 0.000367429 | V19-J2-4 | 0.016721735 | V5-1-J1-3 | 2.20E-25 | V6-1-J1-1 |
| 0.00037583 | V5-4-J2-2 | 0.016994243 | V11-2-J2-7 | 2.84E-25 | V28-J1-3 |
| 0.000402123 | V5-1-J1-2 | 0.016994243 | V5-5-J1-4 | 2.92E-25 | V12-4-J2-5 |
| 0.000411262 | V25-1-J1-6 | 0.018712575 | V5-1-J1-1 | 3.01E-25 | V4-2-J2-3 |
| 0.000411262 | V19-J1-5 | 0.019627385 | V7-8-J2-2 | 4.28E-25 | V6-1-J1-4 |
| 0.000430126 | V25-1-J1-4 | 0.019627385 | V12-5-J2-5 | 4.41E-25 | V6-9-J2-1 |
| 0.000439858 | V19-J2-2 | 0.019940884 | V5-1-J1-4 | 4.66E-25 | V12-4-J1-3 |
| 0.000439858 | V9-J1-1 | 0.019940884 | V20-1-J2-4 | 4.73E-25 | V6-9-J1-1 |
| 0.000470302 | V4-1-J1-2 | 0.020581018 | V19-J2-1 | 5.01E-25 | V6-1-J2-1 |
| 0.000470302 | V12-5-J1-2 | 0.021238998 | V11-2-J1-5 | 5.15E-25 | V4-1-J2-5 |
| 0.000480879 | V4-3-J1-1 | 0.021238998 | V6-9-J2-3 | 5.15E-25 | V6-9-J2-3 |
| 0.000491677 | V18-J1-5 | 0.021574806 | V20-1-J2-3 | 5.15E-25 | V4-3-J1-1 |
| 0.000537173 | V10-1-J1-5 | 0.02226031 | V11-3-J2-4 | 8.08E-25 | V6-1-J2-7 |
| 0.000537173 | V10-3-J1-3 | 0.02226031 | V29-1-J1-1 | 9.05E-25 | V4-2-J1-3 |
| 0.000537173 | V6-5-J2-2 | 0.02261011 | V30-J1-1 | 1.32E-24 | V12-4-J2-2 |
| 0.000549145 | V10-3-J2-4 | 0.02261011 | V5-5-J1-1 | 1.65E-24 | V6-1-J2-5 |
| 0.000561364 | V10-3-J1-2 | 0.022964677 | V10-3-J1-2 | 2.48E-24 | V6-1-J2-4 |
| 0.000573837 | V4-3-J2-3 | 0.02519515 | V6-5-J2-4 | 2.66E-24 | V28-J1-4 |
| 0.000573837 | V11-3-J2-5 | 0.025979194 | V11-2-J1-2 | 3.46E-24 | V4-3-J2-7 |
| 0.000586567 | V18-J1-2 | 0.025979194 | V7-2-J2-1 | 3.61E-24 | V4-2-J1-2 |
| 0.00061282 | V5-1-J1-1 | 0.026379066 | V10-2-J2-4 | 4.20E-24 | V4-1-J1-4 |
| 0.00061282 | V6-1-J2-7 | 0.027194787 | V19-J1-1 | 5.46E-24 | V12-4-J1-4 |
| 0.000640163 | V9-J2-4 | 0.027194787 | V6-1-J1-1 | 6.45E-24 | V12-4-J1-6 |
| 0.000654256 | V27-J1-1 | 0.027610748 | V24-1-J1-3 | 6.54E-24 | V6-9-J1-5 |
| 0.000654256 | V11-3-J1-5 | 0.028032185 | V2-J1-6 | 8.27E-24 | V4-3-J2-1 |
| 0.000668637 | V25-1-J1-2 | 0.028032185 | V19-J2-3 | 1.06E-23 | V4-2-J1-6 |
| 0.000698285 | V11-2-J2-1 | 0.028891715 | V9-J2-5 | 1.06E-23 | V4-2-J1-4 |
| 0.000713562 | V11-2-J1-2 | 0.028891715 | V7-4-J2-4 | 1.12E-23 | V4-1-J1-6 |
| 0.000745054 | V11-2-J1-1 | 0.029329923 | V6-5-J1-2 | 1.13E-23 | V6-9-J2-7 |
| 0.000794719 | V5-1-J1-5 | 0.029773838 | V7-8-J1-4 | 2.16E-23 | V4-1-J2-4 |
| 0.000794719 | V18-J1-4 | 0.030223518 | V5-5-J1-3 | 2.91E-23 | V4-3-J2-5 |
| 0.000829518 | V6-1-J2-5 | 0.030223518 | V5-6-J2-2 | 3.34E-23 | V6-5-J1-5 |
| 0.000847443 | V18-J2-4 | 0.03067902 | V11-3-J1-5 | 4.68E-23 | V4-3-J1-2 |
| 0.000884374 | V19-J1-6 | 0.032081063 | V6-1-J2-3 | 5.08E-23 | V27-J2-6 |
| 0.000884374 | V7-6-J2-5 | 0.03304597 | V5-1-J1-5 | 5.97E-23 | V7-4-J1-2 |
| 0.000884374 | V6-6-J1-1 | 0.033537668 | V5-6-J1-3 | 8.14E-23 | V27-J1-4 |
| 0.000903394 | V5-4-J2-3 | 0.033537668 | V7-9-J2-1 | 8.14E-23 | V6-5-J2-2 |
| 0.000922793 | V5-4-J2-1 | 0.035567524 | V11-2-J2-4 | 9.07E-23 | V4-3-J2-2 |
| 0.000962754 | V25-1-J1-1 | 0.035567524 | V2-J1-4 | 1.08E-22 | V7-4-J2-5 |
| 0.001004312 | V3-1-J2-7 | 0.035567524 | V4-1-J1-5 | 1.17E-22 | V12-4-J2-6 |
| 0.001025708 | V4-3-J2-4 | 0.036091065 | V10-1-J2-5 | 1.23E-22 | V7-4-J2-3 |
| 0.001047525 | V5-4-J1-3 | 0.036621161 | V2-J1-1 | 1.53E-22 | V27-J1-3 |
| 0.001069771 | V3-1-J1-5 | 0.038808371 | V5-4-J1-2 | 1.68E-22 | V6-9-J2-5 |
| 0.001092453 | V27-J2-4 | 0.038808371 | V30-J2-5 | 2.11E-22 | V4-3-J1-4 |
| 0.00111558 | V5-1-J2-4 | 0.039942968 | V12-5-J2-1 | 5.20E-22 | V6-9-J1-2 |
| 0.001163196 | V28-J1-2 | 0.040520741 | V12-5-J2-7 | 9.16E-22 | V10-1-J1-1 |
| 0.001187703 | V19-J1-4 | 0.040520741 | V25-1-J1-4 | 1.07E-21 | V4-3-J2-3 |
| 0.001187703 | V7-6-J2-1 | 0.041697561 | V9-J1-4 | 1.21E-21 | V5-4-J2-2 |
| 0.001238153 | V18-J2-2 | 0.043516969 | V11-2-J2-5 | 1.67E-21 | V4-3-J1-3 |
| 0.001290575 | V11-3-J2-2 | 0.043516969 | V9-J2-2 | 1.69E-21 | V6-9-J2-2 |
| 0.001290575 | V7-2-J2-5 | 0.044766801 | V14-J1-3 | 1.96E-21 | V5-4-J2-1 |
| 0.001317547 | V19-J2-6 | 0.045402987 | V10-3-J1-1 | 2.64E-21 | V5-8-J2-1 |
| 0.001317547 | V3-1-J2-3 | 0.046046775 | V11-1-J1-1 | 3.25E-21 | V5-4-J2-7 |
| 0.001317547 | V3-1-J1-6 | 0.046046775 | V7-9-J2-6 | 3.55E-21 | V7-4-J1-1 |
| 0.001345039 | V18-J2-3 | 0.046046775 | V5-6-J1-6 | 3.84E-21 | V4-2-J2-6 |
| 0.001345039 | V4-3-J2-1 | 0.04802444 | V7-2-J1-2 | 4.04E-21 | V7-4-J2-1 |
| 0.001401617 | V19-J1-1 | 0.048699322 | V6-5-J2-3 | 5.80E-21 | V10-1-J2-3 |
| 0.001490609 | V28-J2-6 |  |  | 9.82E-21 | V4-1-J2-6 |
| 0.001617369 | V5-1-J1-3 |  |  | 1.01E-20 | V6-2-J1-3 |
| 0.001650573 | V28-J1-4 |  |  | 1.03E-20 | V6-2-J1-6 |
| 0.001650573 | V3-1-J2-4 |  |  | 1.13E-20 | V4-3-J1-6 |
| 0.001684403 | V5-6-J2-5 |  |  | 1.40E-20 | V6-1-J2-6 |
| 0.001753984 | V5-4-J1-2 |  |  | 1.52E-20 | V10-1-J2-7 |
| 0.001753984 | V3-1-J1-2 |  |  | 2.34E-20 | V12-4-J2-4 |
| 0.001753984 | V6-5-J1-5 |  |  | 3.25E-20 | V10-1-J2-5 |
| 0.0018262 | V3-1-J1-4 |  |  | 5.44E-20 | V5-4-J2-5 |
| 0.0018262 | V5-1-J2-6 |  |  | 5.86E-20 | V5-8-J2-3 |
| 0.0018262 | V5-4-J1-4 |  |  | 6.09E-20 | V5-8-J2-7 |
| 0.001863323 | V11-2-J1-6 |  |  | 6.48E-20 | V5-8-J1-1 |
| 0.001978892 | V25-1-J2-2 |  |  | 6.82E-20 | V27-J2-4 |
| 0.002018853 | V7-6-J1-5 |  |  | 7.63E-20 | V5-4-J2-3 |
| 0.002059554 | V10-3-J1-5 |  |  | 9.20E-20 | V7-3-J1-5 |
| 0.002143223 | V4-1-J1-3 |  |  | 1.00E-19 | V7-9-J2-2 |
| 0.002143223 | V6-9-J2-7 |  |  | 1.18E-19 | V5-6-J2-7 |
| 0.002186216 | V4-3-J1-6 |  |  | 2.17E-19 | V4-2-J2-4 |
| 0.002274582 | V4-3-J1-3 |  |  | 2.67E-19 | V7-6-J1-2 |
| 0.002319981 | V6-6-J2-2 |  |  | 3.02E-19 | V10-1-J2-1 |
| 0.002319981 | V20-1-J2-5 |  |  | 3.29E-19 | V10-1-J1-5 |
| 0.002366209 | V7-9-J2-3 |  |  | 4.70E-19 | V5-6-J2-3 |
| 0.002461204 | V11-2-J1-5 |  |  | 6.38E-19 | V18-J2-5 |
| 0.002461204 | V7-3-J2-3 |  |  | 7.21E-19 | V6-2-J1-5 |
| 0.002610253 | V4-3-J2-6 |  |  | 8.44E-19 | V7-9-J2-3 |
| 0.002661741 | V9-J1-6 |  |  | 9.53E-19 | V7-4-J2-2 |
| 0.002661741 | V27-J1-5 |  |  | 1.06E-18 | V5-8-J2-2 |
| 0.002821825 | V12-5-J1-1 |  |  | 1.34E-18 | V7-4-J2-7 |
| 0.002877109 | V9-J1-5 |  |  | 1.60E-18 | V10-1-J2-2 |
| 0.002877109 | V10-3-J1-1 |  |  | 1.79E-18 | V5-5-J2-1 |
| 0.002877109 | V7-3-J2-2 |  |  | 1.99E-18 | V7-6-J2-4 |
| 0.002933381 | V12-5-J2-5 |  |  | 2.04E-18 | V10-1-J1-2 |
| 0.003108274 | V4-1-J1-1 |  |  | 2.47E-18 | V18-J2-1 |
| 0.003108274 | V11-3-J2-4 |  |  | 2.89E-18 | V5-6-J2-2 |
| 0.003168652 | V25-1-J2-1 |  |  | 3.03E-18 | V6-5-J2-3 |
| 0.003230097 | V7-8-J2-5 |  |  | 3.18E-18 | V5-6-J2-1 |
| 0.003292626 | V6-1-J1-1 |  |  | 3.89E-18 | V7-3-J1-3 |
| 0.003292626 | V19-J2-1 |  |  | 4.13E-18 | V5-5-J2-7 |
| 0.003356255 | V27-J2-2 |  |  | 4.18E-18 | V16-J2-5 |
| 0.003356255 | V3-1-J1-1 |  |  | 4.23E-18 | V7-3-J1-4 |
| 0.003356255 | V6-5-J2-5 |  |  | 4.39E-18 | V5-4-J1-2 |
| 0.003356255 | V18-J1-3 |  |  | 5.56E-18 | V5-5-J2-2 |
| 0.003421002 | V7-7-J1-1 |  |  | 5.70E-18 | V7-9-J1-5 |
| 0.003421002 | V7-3-J2-5 |  |  | 1.05E-17 | V5-6-J1-1 |
| 0.003486885 | V4-1-J1-6 |  |  | 1.07E-17 | V7-4-J1-6 |
| 0.003486885 | V18-J2-1 |  |  | 1.18E-17 | V7-4-J1-4 |
| 0.00355392 | V5-6-J1-1 |  |  | 1.24E-17 | V6-2-J1-4 |
| 0.00355392 | V7-9-J2-5 |  |  | 1.46E-17 | V6-5-J2-5 |
| 0.003762119 | V15-J1-5 |  |  | 2.62E-17 | V18-J2-7 |
| 0.003833945 | V3-1-J2-2 |  |  | 3.05E-17 | V11-1-J1-2 |
| 0.003833945 | V4-1-J2-3 |  |  | 3.08E-17 | V6-2-J2-3 |
| 0.003907014 | V10-1-J2-5 |  |  | 3.23E-17 | V4-3-J2-4 |
| 0.003907014 | V10-3-J1-6 |  |  | 3.46E-17 | V7-3-J1-2 |
| 0.003981346 | V4-3-J2-2 |  |  | 3.50E-17 | V4-3-J2-6 |
| 0.00405696 | V6-1-J2-3 |  |  | 3.50E-17 | V6-2-J2-5 |
| 0.004133875 | V9-J1-4 |  |  | 4.12E-17 | V11-1-J2-2 |
| 0.004212111 | V12-3-J2-5 |  |  | 4.68E-17 | V6-5-J1-1 |
| 0.004372624 | V4-2-J1-2 |  |  | 7.00E-17 | V5-4-J1-1 |
| 0.004623806 | V10-3-J2-2 |  |  | 8.70E-17 | V16-J2-3 |
| 0.004798445 | V4-1-J1-4 |  |  | 8.80E-17 | V6-2-J1-2 |
| 0.005165742 | V5-6-J1-2 |  |  | 9.32E-17 | V11-1-J1-1 |
| 0.005165742 | V5-4-J1-6 |  |  | 1.14E-16 | V5-5-J2-3 |
| 0.005358757 | V10-3-J1-4 |  |  | 1.30E-16 | V6-9-J1-6 |
| 0.005358757 | V5-1-J1-4 |  |  | 1.39E-16 | V7-8-J1-2 |
| 0.005457686 | V5-6-J2-7 |  |  | 1.72E-16 | V6-2-J2-2 |
| 0.005558261 | V16-J1-2 |  |  | 2.62E-16 | V16-J2-7 |
| 0.005660505 | V7-7-J2-2 |  |  | 2.99E-16 | V5-8-J2-5 |
| 0.005660505 | V4-3-J1-5 |  |  | 3.17E-16 | V6-5-J1-3 |
| 0.005660505 | V7-6-J2-7 |  |  | 3.24E-16 | V6-2-J2-1 |
| 0.005660505 | V11-3-J2-6 |  |  | 3.35E-16 | V18-J2-3 |
| 0.005870098 | V5-4-J1-1 |  |  | 3.58E-16 | V6-2-J1-1 |
| 0.005870098 | V6-6-J2-5 |  |  | 4.01E-16 | V6-5-J1-6 |
| 0.005977496 | V6-1-J1-5 |  |  | 4.38E-16 | V7-6-J1-1 |
| 0.00619762 | V2-J1-2 |  |  | 4.74E-16 | V16-J2-1 |
| 0.006425015 | V6-6-J2-3 |  |  | 6.84E-16 | V5-4-J1-5 |
| 0.006659889 | V27-J1-4 |  |  | 7.32E-16 | V7-3-J2-7 |
| 0.006902455 | V9-J2-5 |  |  | 9.97E-16 | V7-9-J2-1 |
| 0.006902455 | V25-1-J1-3 |  |  | 1.03E-15 | V7-9-J1-3 |
| 0.006902455 | V6-9-J2-1 |  |  | 1.07E-15 | V5-6-J2-5 |
| 0.007152928 | V11-1-J2-5 |  |  | 1.15E-15 | V11-1-J2-3 |
| 0.007152928 | V7-3-J2-7 |  |  | 1.79E-15 | V7-9-J1-6 |
| 0.00741153 | V15-J2-3 |  |  | 2.01E-15 | V18-J1-2 |
| 0.00741153 | V2-J1-1 |  |  | 2.30E-15 | V7-3-J1-1 |
| 0.007543949 | V15-J1-6 |  |  | 2.35E-15 | V5-4-J1-6 |
| 0.007543949 | V7-6-J2-2 |  |  | 2.35E-15 | V11-1-J2-1 |
| 0.007678486 | V28-J2-4 |  |  | 2.37E-15 | V11-1-J2-7 |
| 0.007954029 | V5-4-J2-5 |  |  | 2.59E-15 | V6-5-J2-1 |
| 0.008095094 | V5-4-J2-7 |  |  | 4.75E-15 | V6-5-J2-7 |
| 0.008095094 | V6-1-J2-1 |  |  | 4.80E-15 | V5-5-J1-5 |
| 0.008095094 | V11-1-J2-3 |  |  | 7.62E-15 | V6-5-J1-4 |
| 0.008095094 | V4-1-J2-1 |  |  | 8.39E-15 | V18-J1-1 |
| 0.008383961 | V15-J1-2 |  |  | 8.57E-15 | V5-8-J1-2 |
| 0.008531823 | V2-J1-6 |  |  | 9.24E-15 | V5-8-J1-5 |
| 0.008531823 | V5-1-J2-2 |  |  | 1.04E-14 | V7-4-J1-5 |
| 0.008989499 | V12-5-J2-7 |  |  | 1.12E-14 | V7-3-J1-6 |
| 0.008989499 | V2-J1-3 |  |  | 1.14E-14 | V5-6-J1-3 |
| 0.009468975 | V5-1-J1-6 |  |  | 1.62E-14 | V16-J2-2 |
| 0.009468975 | V18-J1-1 |  |  | 2.47E-14 | V3-1-J2-7 |
| 0.009633796 | V15-J1-3 |  |  | 3.57E-14 | V7-9-J2-7 |
| 0.009633796 | V5-6-J2-4 |  |  | 3.61E-14 | V7-3-J2-2 |
| 0.00997113 | V16-J2-7 |  |  | 3.80E-14 | V7-6-J1-5 |
| 0.010143711 | V5-4-J2-4 |  |  | 4.00E-14 | V7-3-J2-4 |
| 0.010677519 | V5-1-J2-7 |  |  | 4.98E-14 | V5-5-J2-5 |
| 0.011236161 | V16-J2-1 |  |  | 5.09E-14 | V18-J1-5 |
| 0.011236161 | V7-8-J1-6 |  |  | 5.30E-14 | V6-5-J1-2 |
| 0.011428061 | V3-1-J2-1 |  |  | 5.76E-14 | V7-8-J1-3 |
| 0.011820609 | V12-5-J2-3 |  |  | 6.00E-14 | V7-4-J1-3 |
| 0.012021331 | V6-5-J2-7 |  |  | 6.66E-14 | V18-J1-4 |
| 0.012225069 | V10-1-J2-3 |  |  | 6.94E-14 | V11-1-J2-5 |
| 0.01243186 | V6-5-J2-3 |  |  | 7.01E-14 | V5-6-J1-2 |
| 0.012641744 | V3-1-J2-6 |  |  | 7.38E-14 | V7-4-J2-4 |
| 0.013070941 | V2-J2-4 |  |  | 8.18E-14 | V5-5-J1-1 |
| 0.013290334 | V6-1-J1-3 |  |  | 8.79E-14 | V18-J2-6 |
| 0.013290334 | V15-J2-6 |  |  | 1.10E-13 | V16-J1-4 |
| 0.013512976 | V28-J1-1 |  |  | 1.11E-13 | V5-5-J1-6 |
| 0.013968167 | V16-J1-1 |  |  | 1.12E-13 | V5-6-J1-5 |
| 0.013968167 | V24-1-J1-2 |  |  | 1.56E-13 | V5-6-J1-6 |
| 0.014200798 | V11-2-J1-3 |  |  | 1.56E-13 | V7-9-J1-1 |
| 0.014919328 | V5-1-J2-3 |  |  | 1.87E-13 | V11-1-J1-5 |
| 0.014919328 | V2-J2-1 |  |  | 1.87E-13 | V10-1-J1-3 |
| 0.015165857 | V6-8-J1-5 |  |  | 2.18E-13 | V7-3-J2-1 |
| 0.015165857 | V12-3-J1-5 |  |  | 2.67E-13 | V7-8-J1-6 |
| 0.015415966 | V7-2-J2-7 |  |  | 2.70E-13 | V7-9-J2-5 |
| 0.015669698 | V18-J2-6 |  |  | 2.86E-13 | V7-4-J2-6 |
| 0.016188207 | V3-1-J2-5 |  |  | 2.89E-13 | V6-2-J2-7 |
| 0.016453072 | V4-3-J2-7 |  |  | 3.36E-13 | V3-1-J2-1 |
| 0.016453072 | V3-1-J1-3 |  |  | 4.07E-13 | V11-1-J1-4 |
| 0.016994243 | V14-J2-6 |  |  | 5.28E-13 | V11-1-J1-6 |
| 0.01727064 | V7-8-J2-2 |  |  | 6.01E-13 | V11-1-J2-4 |
| 0.01727064 | V27-J1-2 |  |  | 7.70E-13 | V18-J2-2 |
| 0.017550972 | V5-5-J2-1 |  |  | 8.41E-13 | V10-1-J1-6 |
| 0.017550972 | V6-6-J2-1 |  |  | 1.06E-12 | V6-9-J1-3 |
| 0.017550972 | V7-4-J1-6 |  |  | 1.08E-12 | V12-5-J2-3 |
| 0.018123625 | V27-J2-7 |  |  | 1.21E-12 | V7-8-J2-3 |
| 0.018123625 | V4-1-J2-4 |  |  | 1.22E-12 | V5-4-J2-6 |
| 0.018416039 | V5-6-J2-3 |  |  | 1.24E-12 | V24-1-J2-2 |
| 0.018712575 | V2-J2-7 |  |  | 1.28E-12 | V7-9-J2-4 |
| 0.0193182 | V13-J2-2 |  |  | 1.34E-12 | V6-9-J1-4 |
| 0.0193182 | V6-6-J2-7 |  |  | 1.79E-12 | V20-1-J2-7 |
| 0.019940884 | V5-4-J1-5 |  |  | 1.79E-12 | V18-J2-4 |
| 0.019940884 | V7-8-J2-3 |  |  | 2.64E-12 | V7-3-J2-3 |
| 0.020581018 | V12-3-J2-2 |  |  | 3.88E-12 | V3-1-J2-3 |
| 0.020907752 | V19-J1-3 |  |  | 3.99E-12 | V12-5-J2-1 |
| 0.021238998 | V5-6-J2-1 |  |  | 3.99E-12 | V5-4-J1-4 |
| 0.021238998 | V7-2-J1-1 |  |  | 5.03E-12 | V20-1-J2-1 |
| 0.021238998 | V7-2-J2-1 |  |  | 5.07E-12 | V6-5-J2-4 |
| 0.021574806 | V6-8-J2-2 |  |  | 5.17E-12 | V7-6-J1-3 |
| 0.021915226 | V4-2-J2-1 |  |  | 6.75E-12 | V20-1-J2-4 |
| 0.021915226 | V4-2-J1-5 |  |  | 6.82E-12 | V11-1-J2-6 |
| 0.021915226 | V5-5-J1-4 |  |  | 9.31E-12 | V10-1-J2-4 |
| 0.02226031 | V12-5-J1-5 |  |  | 1.10E-11 | V7-8-J1-5 |
| 0.02226031 | V15-J2-1 |  |  | 1.26E-11 | V9-J2-1 |
| 0.022964677 | V4-1-J2-6 |  |  | 1.36E-11 | V16-J1-5 |
| 0.022964677 | V7-9-J2-1 |  |  | 1.39E-11 | V24-1-J2-5 |
| 0.023324063 | V16-J1-5 |  |  | 1.39E-11 | V5-5-J1-2 |
| 0.023324063 | V28-J1-3 |  |  | 1.63E-11 | V16-J2-4 |
| 0.023324063 | V7-2-J1-5 |  |  | 1.81E-11 | V10-3-J2-5 |
| 0.023324063 | V5-5-J2-2 |  |  | 2.04E-11 | V3-1-J2-2 |
| 0.023688323 | V6-2-J1-1 |  |  | 2.06E-11 | V10-3-J2-7 |
| 0.024057507 | V10-1-J2-6 |  |  | 2.10E-11 | V7-6-J2-2 |
| 0.024431671 | V7-6-J2-3 |  |  | 2.20E-11 | V6-2-J2-4 |
| 0.024431671 | V11-1-J2-7 |  |  | 2.85E-11 | V5-4-J2-4 |
| 0.025584574 | V7-2-J2-3 |  |  | 3.43E-11 | V7-9-J1-2 |
| 0.025979194 | V5-1-J2-1 |  |  | 4.19E-11 | V18-J1-3 |
| 0.025979194 | V2-J2-5 |  |  | 4.63E-11 | V5-4-J1-3 |
| 0.026379066 | V2-J1-5 |  |  | 4.63E-11 | V25-1-J2-3 |
| 0.026784245 | V30-J1-2 |  |  | 5.03E-11 | V7-8-J2-4 |
| 0.027610748 | V4-2-J2-6 |  |  | 5.36E-11 | V3-1-J2-5 |
| 0.028891715 | V6-6-J1-5 |  |  | 7.16E-11 | V12-5-J2-5 |
| 0.028891715 | V5-6-J1-4 |  |  | 7.84E-11 | V10-2-J2-1 |
| 0.028891715 | V5-1-J2-5 |  |  | 8.28E-11 | V24-1-J2-4 |
| 0.029329923 | V7-3-J2-1 |  |  | 8.43E-11 | V3-1-J1-2 |
| 0.030223518 | V9-J1-3 |  |  | 1.05E-10 | V7-8-J2-2 |
| 0.030223518 | V6-2-J1-5 |  |  | 1.23E-10 | V3-1-J1-1 |
| 0.031607734 | V5-6-J1-6 |  |  | 1.51E-10 | V11-1-J1-3 |
| 0.031607734 | V2-J2-3 |  |  | 1.69E-10 | V7-6-J1-6 |
| 0.032081063 | V6-6-J1-6 |  |  | 1.95E-10 | V10-2-J1-5 |
| 0.032081063 | V6-1-J2-2 |  |  | 2.39E-10 | V10-3-J2-1 |
| 0.032081063 | V2-J1-4 |  |  | 2.47E-10 | V24-1-J1-5 |
| 0.03304597 | V12-3-J2-4 |  |  | 2.54E-10 | V9-J2-7 |
| 0.03304597 | V6-9-J2-3 |  |  | 2.97E-10 | V24-1-J2-1 |
| 0.03304597 | V6-5-J2-1 |  |  | 3.05E-10 | V5-6-J2-4 |
| 0.033537668 | V10-2-J1-3 |  |  | 3.13E-10 | V7-3-J2-5 |
| 0.03403561 | V15-J1-1 |  |  | 3.13E-10 | V3-1-J1-6 |
| 0.03453986 | V6-2-J1-2 |  |  | 3.57E-10 | V5-5-J1-4 |
| 0.036091065 | V16-J2-3 |  |  | 3.66E-10 | V9-J2-3 |
| 0.036091065 | V7-6-J2-4 |  |  | 3.66E-10 | V7-9-J1-4 |
| 0.036091065 | V14-J1-3 |  |  | 4.67E-10 | V10-2-J2-3 |
| 0.037157876 | V10-1-J2-2 |  |  | 4.71E-10 | V24-1-J1-1 |
| 0.037157876 | V6-1-J1-6 |  |  | 6.32E-10 | V7-6-J2-3 |
| 0.037157876 | V7-9-J1-4 |  |  | 8.03E-10 | V5-5-J1-3 |
| 0.037701274 | V5-4-J2-6 |  |  | 8.67E-10 | V5-5-J2-4 |
| 0.037701274 | V6-1-J1-4 |  |  | 8.74E-10 | V10-1-J1-4 |
| 0.037701274 | V7-2-J2-2 |  |  | 8.97E-10 | V7-6-J1-4 |
| 0.038251417 | V16-J2-4 |  |  | 9.12E-10 | V7-9-J2-6 |
| 0.038251417 | V4-1-J2-5 |  |  | 9.12E-10 | V18-J1-6 |
| 0.038251417 | V4-1-J1-5 |  |  | 9.36E-10 | V5-6-J1-4 |
| 0.0393722 | V20-1-J1-1 |  |  | 9.60E-10 | V5-6-J2-6 |
| 0.040520741 | V10-1-J1-1 |  |  | 1.21E-09 | V24-1-J2-3 |
| 0.041697561 | V30-J1-1 |  |  | 1.45E-09 | V10-3-J1-1 |
| 0.042296741 | V6-9-J2-5 |  |  | 1.85E-09 | V9-J1-2 |
| 0.042296741 | V12-3-J2-1 |  |  | 1.88E-09 | V7-8-J1-1 |
| 0.043516969 | V10-2-J1-1 |  |  | 2.10E-09 | V10-2-J1-6 |
| 0.046698235 | V30-J2-2 |  |  | 2.32E-09 | V9-J2-6 |
| 0.047357434 | V7-4-J2-3 |  |  | 2.56E-09 | V7-8-J2-7 |
| 0.04802444 | V28-J2-3 |  |  | 2.65E-09 | V30-J2-2 |
| 0.048699322 | V7-7-J2-7 |  |  | 3.05E-09 | V7-8-J1-4 |
| 0.048699322 | V30-J1-4 |  |  | 3.45E-09 | V10-3-J2-6 |
| 0.04938215 | V12-3-J1-6 |  |  | 5.28E-09 | V9-J2-2 |
|  |  |  |  | 5.37E-09 | V7-8-J2-1 |
|  |  |  |  | 5.64E-09 | V12-5-J2-2 |
|  |  |  |  | 5.92E-09 | V25-1-J1-1 |
|  |  |  |  | 6.16E-09 | V20-1-J2-6 |
|  |  |  |  | 7.54E-09 | V19-J2-7 |
|  |  |  |  | 8.44E-09 | V25-1-J2-1 |
|  |  |  |  | 1.13E-08 | V19-J2-2 |
|  |  |  |  | 1.14E-08 | V12-5-J2-7 |
|  |  |  |  | 1.16E-08 | V10-3-J1-5 |
|  |  |  |  | 1.32E-08 | V6-8-J2-1 |
|  |  |  |  | 1.39E-08 | V15-J2-1 |
|  |  |  |  | 1.53E-08 | V3-1-J1-5 |
|  |  |  |  | 1.57E-08 | V10-3-J2-3 |
|  |  |  |  | 1.60E-08 | V6-9-J2-4 |
|  |  |  |  | 1.66E-08 | V25-1-J1-4 |
|  |  |  |  | 1.77E-08 | V3-1-J2-4 |
|  |  |  |  | 1.81E-08 | V10-3-J1-2 |
|  |  |  |  | 1.90E-08 | V6-5-J2-6 |
|  |  |  |  | 1.96E-08 | V9-J2-5 |
|  |  |  |  | 2.13E-08 | V6-9-J2-6 |
|  |  |  |  | 2.45E-08 | V7-6-J2-1 |
|  |  |  |  | 2.45E-08 | V16-J2-6 |
|  |  |  |  | 2.61E-08 | V25-1-J1-2 |
|  |  |  |  | 3.12E-08 | V30-J2-6 |
|  |  |  |  | 3.22E-08 | V9-J1-1 |
|  |  |  |  | 3.40E-08 | V30-J1-1 |
|  |  |  |  | 3.76E-08 | V25-1-J1-6 |
|  |  |  |  | 3.76E-08 | V24-1-J1-2 |
|  |  |  |  | 3.90E-08 | V30-J1-2 |
|  |  |  |  | 3.96E-08 | V20-1-J1-4 |
|  |  |  |  | 4.25E-08 | V3-1-J1-3 |
|  |  |  |  | 4.31E-08 | V24-1-J2-7 |
|  |  |  |  | 5.26E-08 | V7-8-J2-5 |
|  |  |  |  | 5.81E-08 | V10-2-J2-7 |
|  |  |  |  | 5.85E-08 | V20-1-J2-5 |
|  |  |  |  | 6.26E-08 | V5-8-J1-6 |
|  |  |  |  | 6.76E-08 | V25-1-J2-2 |
|  |  |  |  | 7.68E-08 | V10-2-J1-1 |
|  |  |  |  | 7.80E-08 | V25-1-J1-3 |
|  |  |  |  | 7.97E-08 | V24-1-J1-4 |
|  |  |  |  | 1.04E-07 | V9-J1-5 |
|  |  |  |  | 1.08E-07 | V7-6-J2-7 |
|  |  |  |  | 1.22E-07 | V6-2-J2-6 |
|  |  |  |  | 1.34E-07 | V9-J1-3 |
|  |  |  |  | 1.44E-07 | V19-J1-1 |
|  |  |  |  | 1.47E-07 | V25-1-J2-7 |
|  |  |  |  | 1.47E-07 | V30-J1-5 |
|  |  |  |  | 1.78E-07 | V30-J2-7 |
|  |  |  |  | 2.20E-07 | V20-1-J1-6 |
|  |  |  |  | 2.36E-07 | V30-J2-1 |
|  |  |  |  | 2.39E-07 | V5-8-J1-4 |
|  |  |  |  | 2.52E-07 | V20-1-J1-1 |
|  |  |  |  | 2.81E-07 | V19-J2-3 |
|  |  |  |  | 3.08E-07 | V15-J2-3 |
|  |  |  |  | 3.41E-07 | V20-1-J2-3 |
|  |  |  |  | 3.71E-07 | V6-8-J1-5 |
|  |  |  |  | 3.99E-07 | V15-J1-6 |
|  |  |  |  | 4.05E-07 | V9-J1-4 |
|  |  |  |  | 4.07E-07 | V25-1-J2-5 |
|  |  |  |  | 4.25E-07 | V19-J2-1 |
|  |  |  |  | 4.41E-07 | V30-J2-3 |
|  |  |  |  | 4.44E-07 | V20-1-J1-2 |
|  |  |  |  | 5.33E-07 | V10-3-J1-4 |
|  |  |  |  | 5.33E-07 | V24-1-J2-6 |
|  |  |  |  | 5.60E-07 | V5-1-J2-5 |
|  |  |  |  | 6.01E-07 | V24-1-J1-6 |
|  |  |  |  | 6.49E-07 | V15-J2-5 |
|  |  |  |  | 7.78E-07 | V10-3-J1-6 |
|  |  |  |  | 7.84E-07 | V10-3-J2-2 |
|  |  |  |  | 8.70E-07 | V15-J2-6 |
|  |  |  |  | 9.45E-07 | V12-5-J1-1 |
|  |  |  |  | 1.01E-06 | V12-5-J1-5 |
|  |  |  |  | 1.25E-06 | V16-J1-2 |
|  |  |  |  | 1.33E-06 | V10-2-J2-4 |
|  |  |  |  | 2.18E-06 | V10-2-J2-2 |
|  |  |  |  | 2.46E-06 | V30-J1-4 |
|  |  |  |  | 2.98E-06 | V30-J2-5 |
|  |  |  |  | 3.10E-06 | V25-1-J1-5 |
|  |  |  |  | 3.33E-06 | V7-3-J2-6 |
|  |  |  |  | 4.03E-06 | V30-J2-4 |
|  |  |  |  | 4.50E-06 | V10-2-J2-5 |
|  |  |  |  | 5.05E-06 | V9-J1-6 |
|  |  |  |  | 5.39E-06 | V11-2-J2-4 |
|  |  |  |  | 5.57E-06 | V5-5-J2-6 |
|  |  |  |  | 5.64E-06 | V10-3-J2-4 |
|  |  |  |  | 6.13E-06 | V20-1-J2-2 |
|  |  |  |  | 6.70E-06 | V3-1-J2-6 |
|  |  |  |  | 6.70E-06 | V24-1-J1-3 |
|  |  |  |  | 7.05E-06 | V20-1-J1-5 |
|  |  |  |  | 7.05E-06 | V10-3-J1-3 |
|  |  |  |  | 7.37E-06 | V19-J2-5 |
|  |  |  |  | 7.96E-06 | V11-2-J1-4 |
|  |  |  |  | 9.44E-06 | V15-J2-7 |
|  |  |  |  | 9.74E-06 | V30-J1-3 |
|  |  |  |  | 9.86E-06 | V11-2-J2-6 |
|  |  |  |  | 1.10E-05 | V19-J1-5 |
|  |  |  |  | 1.12E-05 | V15-J1-5 |
|  |  |  |  | 1.51E-05 | V7-8-J2-6 |
|  |  |  |  | 1.61E-05 | V19-J2-6 |
|  |  |  |  | 1.68E-05 | V12-5-J1-2 |
|  |  |  |  | 2.00E-05 | V5-8-J2-6 |
|  |  |  |  | 2.35E-05 | V7-6-J2-6 |
|  |  |  |  | 2.84E-05 | V7-6-J2-5 |
|  |  |  |  | 2.89E-05 | V10-2-J1-2 |
|  |  |  |  | 3.00E-05 | V19-J2-4 |
|  |  |  |  | 3.13E-05 | V25-1-J2-6 |
|  |  |  |  | 3.78E-05 | V16-J1-1 |
|  |  |  |  | 3.91E-05 | V5-8-J2-4 |
|  |  |  |  | 4.15E-05 | V6-8-J2-7 |
|  |  |  |  | 4.48E-05 | V10-1-J2-6 |
|  |  |  |  | 5.06E-05 | V11-2-J2-5 |
|  |  |  |  | 5.09E-05 | V16-J1-6 |
|  |  |  |  | 6.41E-05 | V7-7-J2-5 |
|  |  |  |  | 7.02E-05 | V3-1-J1-4 |
|  |  |  |  | 7.51E-05 | V7-2-J1-5 |
|  |  |  |  | 8.61E-05 | V11-2-J2-1 |
|  |  |  |  | 8.70E-05 | V11-2-J1-3 |
|  |  |  |  | 8.80E-05 | V19-J1-6 |
|  |  |  |  | 0.000102981 | V5-1-J2-7 |
|  |  |  |  | 0.000113194 | V19-J1-3 |
|  |  |  |  | 0.000119641 | V5-8-J1-3 |
|  |  |  |  | 0.000134322 | V11-2-J2-3 |
|  |  |  |  | 0.000157404 | V15-J1-2 |
|  |  |  |  | 0.000184183 | V11-2-J1-6 |
|  |  |  |  | 0.000187185 | V30-J1-6 |
|  |  |  |  | 0.000214055 | V11-2-J1-1 |
|  |  |  |  | 0.000274639 | V11-2-J2-7 |
|  |  |  |  | 0.000297136 | V7-7-J2-1 |
|  |  |  |  | 0.000345604 | V20-1-J1-3 |
|  |  |  |  | 0.000375417 | V5-1-J2-3 |
|  |  |  |  | 0.000437897 | V6-8-J1-1 |
|  |  |  |  | 0.000460795 | V5-1-J2-1 |
|  |  |  |  | 0.000475065 | V25-1-J2-4 |
|  |  |  |  | 0.00064547 | V6-8-J2-3 |
|  |  |  |  | 0.000674964 | V15-J2-4 |
|  |  |  |  | 0.000702228 | V6-6-J2-4 |
|  |  |  |  | 0.000734121 | V19-J1-2 |
|  |  |  |  | 0.000741389 | V7-2-J1-1 |
|  |  |  |  | 0.000745047 | V15-J1-4 |
|  |  |  |  | 0.000846272 | V5-1-J2-6 |
|  |  |  |  | 0.000850408 | V9-J2-4 |
|  |  |  |  | 0.00093266 | V6-4-J2-3 |
|  |  |  |  | 0.001027159 | V15-J1-1 |
|  |  |  |  | 0.001119707 | V11-2-J1-2 |
|  |  |  |  | 0.001255114 | V11-3-J1-2 |
|  |  |  |  | 0.001315874 | V11-3-J1-6 |
|  |  |  |  | 0.001459216 | V7-2-J1-3 |
|  |  |  |  | 0.001725131 | V6-4-J2-1 |
|  |  |  |  | 0.001765401 | V10-2-J1-3 |
|  |  |  |  | 0.001773557 | V15-J2-2 |
|  |  |  |  | 0.001935272 | V5-1-J1-2 |
|  |  |  |  | 0.002158875 | V10-2-J1-4 |
|  |  |  |  | 0.002561754 | V6-6-J2-7 |
|  |  |  |  | 0.002992492 | V12-5-J1-6 |
|  |  |  |  | 0.002992492 | V16-J1-3 |
|  |  |  |  | 0.003113452 | V6-6-J1-5 |
|  |  |  |  | 0.003458122 | V7-2-J2-2 |
|  |  |  |  | 0.003611717 | V11-2-J2-2 |
|  |  |  |  | 0.00375523 | V11-3-J1-3 |
|  |  |  |  | 0.003903908 | V6-4-J2-2 |
|  |  |  |  | 0.004217413 | V11-2-J1-5 |
|  |  |  |  | 0.004670926 | V6-6-J1-1 |
|  |  |  |  | 0.004790996 | V6-6-J1-4 |
|  |  |  |  | 0.004852072 | V6-6-J2-5 |
|  |  |  |  | 0.004934603 | V13-J2-7 |
|  |  |  |  | 0.005344228 | V6-6-J1-6 |
|  |  |  |  | 0.005434283 | V14-J1-3 |
|  |  |  |  | 0.005760364 | V19-J1-4 |
|  |  |  |  | 0.008049901 | V6-6-J2-1 |
|  |  |  |  | 0.008180018 | V12-5-J1-3 |
|  |  |  |  | 0.009626268 | V6-4-J2-6 |
|  |  |  |  | 0.009972727 | V12-5-J2-4 |
|  |  |  |  | 0.012436485 | V6-6-J1-2 |
|  |  |  |  | 0.014257335 | V11-3-J1-5 |
|  |  |  |  | 0.016430703 | V6-6-J2-6 |
|  |  |  |  | 0.017687516 | V7-7-J2-7 |
|  |  |  |  | 0.018414059 | V14-J2-6 |
|  |  |  |  | 0.019097085 | V13-J1-6 |
|  |  |  |  | 0.020529853 | V5-1-J1-5 |
|  |  |  |  | 0.020977574 | V11-3-J1-4 |
|  |  |  |  | 0.021204609 | V2-J2-2 |
|  |  |  |  | 0.022372169 | V7-2-J1-6 |
|  |  |  |  | 0.022854615 | V14-J1-2 |
|  |  |  |  | 0.023678787 | V6-4-J1-3 |
|  |  |  |  | 0.026034057 | V7-2-J1-4 |
|  |  |  |  | 0.02908534 | V6-6-J2-3 |
|  |  |  |  | 0.031894537 | V11-3-J2-7 |
|  |  |  |  | 0.032220577 | V6-8-J1-3 |
|  |  |  |  | 0.037093532 | V6-6-J1-3 |
|  |  |  |  | 0.038979723 | V5-1-J1-1 |
|  |  |  |  | 0.044988752 | V14-J2-3 |
|  |  |  |  | 0.047662988 | V11-3-J2-4 |
|  |  |  |  | 0.04904945 | V12-5-J1-4 |
|  |  |  |  | 0.049519062 | V29-1-J2-7 |
