# Supplementary material for: T cell receptor repertoire as a novel indicator for identification and immune surveillance of patients with severe obstructive sleep apnea
Source: PeerJ. 2023 Apr 7;11:e15009. doi: 10.7717/peerj.15009 (PMC10084822; doi:10.7717/peerj.15009)
Supplement: Supplemental Information 3 [file peerj-11-15009-s003.docx]

| **TableS3: Unique CDR3 Sequences Between Groups** | | | | | | |
| --- | --- | --- | --- | --- | --- | --- |
| Group | Compared to | CDR3 | FC | PValue | sig | Lablel |
| Severe OSA | mOSA | ASSLGGNQPQH | 3.057381909 | 0.002059554 | Up |  |
|  |  | ASSLGTANTGELF | 0.169570137 | 0.042903188 | Down |  |
|  |  | ASSLGGSGANVLT | 8.501407452 | 0.044766801 | Up |  |
|  |  | ASSESTDTQY | 0.03301437 | 0.04938215 | Down |  |
|  |  | ASSPTGTDTQY | 0.10245048 | 0.03453986 | Down |  |
|  |  | ASSLGPYNEQF | 9.879822196 | 0.016188207 | Up |  |
|  |  | ASSLGNTGELF | 0.061960451 | 0.019940884 | Down |  |
|  |  | ASSGTGGNQPQH | 0.23014565 | 0.042903188 | Down |  |
|  |  | ASSLQGSTDTQY | 0.145363815 | 0.031607734 | Down |  |
|  | Non-OSA | ASSLGGEETQY | 0.160078952 | 0.03067902 | Down |  |
|  |  | ASSLGYEQY | 0.500595935 | 0.048699322 | Down |  |
|  |  | ASSLDYEQY | 0.328640268 | 0.044138151 | Down |  |
|  |  | ASSLRETQY | 0.592905221 | 0.03453986 | Down |  |
|  |  | ASSSSQETQY | 0.230093143 | 0.042296741 | Down |  |
|  |  | ASSLIGVSSYNEQF | 0.01090027 | 0.043516969 | Down |  |
|  |  | ASSLAGGQETQY | 0.297740643 | 0.041697561 | Down |  |
|  |  | ASSPTNTGELF | 0.189520628 | 0.03403561 | Down |  |
|  |  | ASSLGGNEQF | 0.575045519 | 0.040520741 | Down |  |
|  |  | ASSSGSSYNEQF | 0.020612621 | 0.004979032 | Down | ASSSGSSYNEQF |
|  | HD | ASSLGGNQPQH | 2.289045474 | 5.61E-05 | Up |  |
|  |  | ASSLTANTGELF | 6.795976429 | 0.030828086 | Up |  |
|  |  | ASSFVSGTDTQY | 74.21480898 | 0.038723616 | Up |  |
|  |  | ASSYSSNQPQH | 23.79291582 | 0.002812832 | Up |  |
|  |  | ASSLGTSTDTQY | 1.744208032 | 0.036725769 | Up |  |
|  |  | ASSPQETQY | 0.369425541 | 0.032770323 | Down |  |
|  |  | ASSLGQGAYEQY | 1.537187899 | 0.024442467 | Up |  |
|  |  | ASSLLSTDTQY | 4.762173886 | 0.046161262 | Up |  |
|  |  | ASSLGGNTEAF | 2.592584452 | 0.045865748 | Up |  |
|  |  | ASSLQGYEQY | 1.865215668 | 0.037093532 | Up |  |
|  |  | ASSLTGGTEAF | 2.753748225 | 0.026862916 | Up |  |
|  |  | ASSLGPYNEQF | 1.753330537 | 0.012341653 | Up |  |
|  |  | ASSLSSGNTIY | 1.982711415 | 0.038468941 | Up |  |
|  |  | ASSLAGYEQY | 1.614765729 | 0.034467999 | Up |  |
|  |  | ASSFGNEQF | 19.87676723 | 0.032549468 | Up |  |
|  |  | ASSLGGSGNTIY | 2.092897368 | 0.04067973 | Up |  |
|  |  | ASSLGNQPQH | 0.232906612 | 0.036970597 | Down |  |
|  |  | ASSELAGSYNEQF | 9.503344097 | 0.004553586 | Up |  |
|  |  | ASSSSGSTDTQY | 0.040907435 | 0.039887457 | Down |  |
|  |  | ASSHRDRNYEQY | 34.40878872 | 0.013319429 | Up |  |
|  |  | ASSFGQQETQY | 2.425263435 | 0.049519062 | Up |  |
|  |  | ASSLGPTDTQY | 5.882875144 | 0.027523244 | Up |  |
|  |  | ASSLGNTEAF | 2.139746004 | 0.013319429 | Up |  |
|  |  | SARGPEK | 0.000266047 | 0.017687516 | Down |  |
|  |  | ASSLGGDTQY | 1.534999017 | 0.016430703 | Up |  |
|  |  | ASSLGGSSYNEQF | 2.246827239 | 0.034699936 | Up |  |
|  |  | ASSLYNEQF | 0.550567182 | 0.032549468 | Down |  |
|  |  | ASSLTGGTDTQY | 2.555209757 | 0.007547768 | Up |  |
|  |  | ASRPSGGSYEQY | 34.75921199 | 0.035999553 | Up |  |
|  |  | ASSRTYEQY | 2.301935744 | 0.030203027 | Up |  |
|  |  | ASSQDAGNTEAF | 102.2510319 | 0.045865748 | Up |  |
|  |  | ASRGPEK | 0.00011893 | 0.000252408 | Down | ASRGPEK |
|  |  | ASSLTGAYEQY | 8.365964994 | 0.038851491 | Up |  |
|  |  | ASSLVGGNTEAF | 2.119027985 | 0.028295005 | Up |  |
| mOSA | Non-OSA | ASSFTNTGELF | 0.000164268 | 0.043262728 | Down |  |
|  |  | ASSLGGEETQY | 1.00E-04 | 0.01152199 | Down |  |
|  |  | ASSLGYEQY | 0.500011029 | 0.048016062 | Down |  |
|  |  | ASSLDYEQY | 0.190560539 | 0.037885853 | Down |  |
|  |  | ASSLRETQY | 0.444466419 | 0.031320274 | Down |  |
|  |  | ASSSSQETQY | 0.000114991 | 0.043262728 | Down |  |
|  |  | ASSLIGVSSYNEQF | 1.36E-05 | 0.043262728 | Down |  |
|  |  | ASSLGETQY | 0.357148571 | 0.00094516 | Down |  |
|  |  | ASSRDRNTEAF | 17.96188386 | 0.036881613 | Up |  |
|  |  | ASSFGETQY | 0.102576989 | 0.030467091 | Down |  |
|  | HD | ASSLDRNYGYT | 1.975493383 | 0.049459089 | Up |  |
|  |  | ASSFVSGTDTQY | 87.38681782 | 0.020794935 | Up |  |
|  |  | ASSSGQGNSPLH | 2.923869597 | 0.0397115 | Up |  |
|  |  | ASSLSNQPQH | 3.411828121 | 0.025305834 | Up |  |
|  |  | ASSSTYNEQF | 2.408445762 | 0.02115203 | Up |  |
|  |  | ASSLDMNTEAF | 2.861568548 | 0.031139201 | Up |  |
|  |  | ASSLTGGTEAF | 1.902234982 | 0.043129021 | Up |  |
|  |  | ASSYSGNTEAF | 6.817844761 | 0.046555493 | Up |  |
|  |  | ASSELAGGYNEQF | 12.95123055 | 0.038691819 | Up |  |
|  |  | ASSLSGGYEQY | 6.419639004 | 0.013025116 | Up |  |
|  |  | ASSLGQGNTEAF | 1.851918756 | 0.007405363 | Up |  |
|  |  | ASSLGTANTGELF | 4.182042402 | 0.009711984 | Up |  |
|  |  | ASSGTSGTDTQY | 3.232777582 | 0.022255555 | Up |  |
|  |  | ASSQDRGTEAF | 13.61188917 | 0.022006092 | Up |  |
|  |  | ASSPTGTDTQY | 3.836812767 | 0.032853836 | Up |  |
|  |  | ASSLGNTGELF | 11.0879216 | 0.01233671 | Up |  |
|  |  | ASSLAGGNYGYT | 7.945459907 | 0.036334989 | Up |  |
|  |  | ASSPRDSSYEQY | 4.814836715 | 0.049957493 | Up |  |
|  |  | ASSRDTNYGYT | 130.5317322 | 0.042908372 | Up |  |
|  |  | ASSGTGGNQPQH | 4.093162457 | 0.016512589 | Up |  |
|  |  | ASSQGLAGAYEQY | 20.37729799 | 0.02644849 | Up |  |
|  |  | ASRGPEK | 0.00011893 | 0.007793119 | Down | ASRGPEK |
|  |  | ASSLQGSTDTQY | 4.321519058 | 0.036719081 | Up |  |
| Non-OSA | HD | ASSLGGNQPQH | 1.541320068 | 0.03909699 | Up |  |
|  |  | ASSLELNTEAF | 3.324598186 | 0.034648674 | Up |  |
|  |  | ASSLDYEQY | 2.388769296 | 0.028245969 | Up |  |
|  |  | ASSLRETQY | 0.529610763 | 0.046791851 | Down |  |
|  |  | ASSSSQETQY | 7.180338278 | 0.030972039 | Up |  |
|  |  | ASSQDSGSGANVLT | 2609.434783 | 0.043573195 | Up |  |
|  |  | ASSLGQGNYGYT | 3.002146581 | 0.042469937 | Up |  |
|  |  | ASSYGTSTDTQY | 5.522580582 | 0.040125712 | Up |  |
|  |  | ASSSYEQY | 5.559472726 | 0.0482313 | Up |  |
|  |  | ASSLRGTGELF | 4.547592217 | 0.017503356 | Up |  |
|  |  | ASSLGADTQY | 1.691314047 | 0.029183525 | Up |  |
|  |  | ASSPTNTGELF | 2.231179847 | 0.025166051 | Up |  |
|  |  | ASSELAGSYNEQF | 160.5698595 | 0.004439005 | Up | ASSELAGSYNEQF |
|  |  | ASSPPSTDTQY | 0.131327511 | 0.025026936 | Down |  |
|  |  | ASSELAGGPDTQY | 2.181329837 | 0.026303262 | Up |  |
|  |  | ASSLGGSGELF | 11.69011835 | 0.045620086 | Up |  |
|  |  | ASSFTNTGELF | 3.979856444 | 0.024477116 | Up |  |
|  |  | ASSLGGEETQY | 3.567272779 | 0.019530164 | Up |  |
|  |  | ASSLVADTQY | 2.47110384 | 0.049707767 | Up |  |
|  |  | ASSLQAGANEQF | 47.41854621 | 0.049459089 | Up |  |
|  |  | ASSLAGETQY | 2.22401758 | 0.023408808 | Up |  |
|  |  | ASSLGGDTQY | 6.825252771 | 0.049957493 | Up |  |
|  |  | ASSLIGVSSYNEQF | 57.63627862 | 0.009591919 | Up | ASSLIGVSSYNEQF |
|  |  | ASSLDRDTEAF | 2.669816323 | 0.031986671 | Up |  |
|  |  | ASSEASGSSYEQY | 15.48160731 | 0.024888488 | Up |  |
|  |  | ASSLGETQY | 0.321227136 | 0.025166051 | Down |  |
|  |  | ASSYSYEQY | 2.61400615 | 0.02163646 | Up |  |
|  |  | ASSEGTGGADTQY | 12.26939921 | 0.03410163 | Up |  |
|  |  | ASSSGSSYNEQF | 10.63445894 | 0.015118791 | Up |  |
|  |  | ASSLAGGNTEAF | 3.298686554 | 0.025729223 | Up |  |
|  |  | ASSYGSYEQY | 21.77858457 | 0.027185063 | Up |  |
|  |  | ASRGPEK | 0.00011893 | 0.007793119 | Down | ASRGPEK |
