# Supplementary material for: T cell receptor repertoire as a novel indicator for identification and immune surveillance of patients with severe obstructive sleep apnea
Source: PeerJ. 2023 Apr 7;11:e15009. doi: 10.7717/peerj.15009 (PMC10084822; doi:10.7717/peerj.15009)
Supplement: Supplemental Information 4 [file peerj-11-15009-s004.docx]

**TableS4: The AUCs of ROCs by Different Indicators in 96 PSG Participants**

| **Test Varialbes** | **Area** | Std.Ea | Sig.b | **95% CI** | |
| --- | --- | --- | --- | --- | --- |
|  |  |  |  | **Lower limit** | **Upper Limit** |
| OSA-TCI | 0.914 | 0.031 | 0.000 | 0.853 | 0.975 |
| BMI | 0.769 | 0.048 | 0.000 | 0.674 | 0.863 |
| ESS | 0.567 | 0.059 | 0.256 | 0.452 | 0.683 |
| NLR | 0.522 | 0.059 | 0.708 | 0.406 | 0.639 |
| PLR | 0.454 | 0.059 | 0.441 | 0.339 | 0.570 |
| CD4/CD8 | 0.494 | 0.060 | 0.918 | 0.377 | 0.611 |
| a. Assumed nonparametric | | | | | |
| b. Null hypothesis: true region = 0.5 | | | | | |
