# Supplementary material for: T cell receptor repertoire as a novel indicator for identification and immune surveillance of patients with severe obstructive sleep apnea
Source: PeerJ. 2023 Apr 7;11:e15009. doi: 10.7717/peerj.15009 (PMC10084822; doi:10.7717/peerj.15009)
Supplement: Supplemental Information 5 [file peerj-11-15009-s005.docx]

**TableS5: Spearman Correlation Analysis of OSA-TCI Related Clinical Features**

| Variables | correlation coefficient | P value |
| --- | --- | --- |
| OSA-TCI | 1.000 |  |
| Age | -0.013 | 0.902 |
| AHI | .603** | **0.000** |
| D50 | 0.060 | 0.558 |
| ESS | 0.091 | 0.377 |
| BMI | .342** | **0.001** |
| Total White Blood Cell:WBC | 0.108 | 0.297 |
| Neutrophil | 0.033 | 0.748 |
| Lymphocyte | 0.194 | 0.058 |
| Monocyte | 0.058 | 0.574 |
| Eosinophile granulocyte | 0.049 | 0.637 |
| Basophile granulocyte | 0.041 | 0.694 |
| Leukocyte-to-lymphocyte ratio:LLR | -0.083 | 0.424 |
| Neutrophil-to-lymphocyte ratio:NLR | -0.084 | 0.414 |
| Platelet-to-lymphocyte ratio:PLR | -0.082 | 0.426 |
| HGB | .307** | **0.002** |
| PLT | 0.091 | 0.378 |
| CD3+T | 0.198 | 0.054 |
| CD4+T | 0.145 | 0.157 |
| CD8+T | 0.153 | 0.136 |
| CD4/CD8 | -0.032 | 0.760 |
| IgG | 0.039 | 0.704 |
| IgA | 0.037 | 0.720 |
| IgM | 0.103 | 0.316 |
| Snoring | .232* | **0.023** |
| Short of breath | 0.137 | 0.183 |
| Arm and Leg movement | 0.146 | 0.156 |
| Increased nocturia | -0.026 | 0.803 |
| Nocturnal Sweating | 0.007 | 0.948 |
| Excessive daytime sleepiness | 0.036 | 0.731 |
| Headache in the morning | 0.029 | 0.781 |
| Thirsty in the morning | 0.062 | 0.547 |
| Moter vechicle accident | -0.094 | 0.362 |
| Work ability impairment | -0.118 | 0.252 |
| Industrial accident history | -0.003 | 0.980 |
| Memory decline | -0.035 | 0.734 |
| Irascibility | -0.118 | 0.253 |
| Achohol | 0.058 | 0.577 |
| Smoking | .319** | **0.002** |
| Total sleep time:TST | 0.009 | 0.928 |
| Sleep efficiency | -0.048 | 0.644 |
| REM duration | -0.057 | 0.580 |
| NREM duration | 0.023 | 0.827 |
| SWS duration | -0.082 | 0.427 |
| Sleep latency | -0.004 | 0.973 |
| Wake time | 0.165 | 0.107 |
| REM proportion（%TST) | -0.024 | 0.814 |
| N1 proportion（%TST) | .369** | **0.000** |
| N2 proportion（%TST) | -.392** | **0.000** |
| N3proportion（%TST) | -0.085 | 0.408 |
| MicroArousal Index | .495** | **0.000** |
| Oxygen Desaturation Index:ODI | .571** | **0.000** |
| Maximal Oxygen desaturation | .535** | **0.000** |
| Leg movements(Index) | 0.119 | 0.248 |
