# Supplementary material for: T cell receptor repertoire as a novel indicator for identification and immune surveillance of patients with severe obstructive sleep apnea
Source: PeerJ. 2023 Apr 7;11:e15009. doi: 10.7717/peerj.15009 (PMC10084822; doi:10.7717/peerj.15009)
Supplement: Supplemental Information 6 [file peerj-11-15009-s006.docx]

**TableS6: Partial Correlation Analysis of OSA-TCI Related Factors Controlled by AHI**

| Controlled Variable | Correlate With |  | OSA-TCI | Smoking | BMI | HGB | N1 （%TST) | N2 （%TST) | MicroArousal | ODI | Lowest SpO_2_ |
| --- | --- | --- | --- | --- | --- | --- | --- | --- | --- | --- | --- |
| AHI指数 | OSA-TCI | R Value | 1.000 | **0.220** | 0.030 | -0.005 | -0.069 | 0.046 | -0.022 | -0.080 | -0.038 |
|  |  | Sig.（two tails） |  | **0.032** | 0.772 | 0.963 | 0.506 | 0.660 | 0.831 | 0.438 | 0.718 |
|  |  | variance | 0 | 93 | 93 | 93 | 93 | 93 | 93 | 93 | 93 |
