# Supplementary material for: T cell receptor repertoire as a novel indicator for identification and immune surveillance of patients with severe obstructive sleep apnea
Source: PeerJ. 2023 Apr 7;11:e15009. doi: 10.7717/peerj.15009 (PMC10084822; doi:10.7717/peerj.15009)
Supplement: Supplemental Information 7 [file peerj-11-15009-s007.docx]

**TableS7: Paired Comparison of Indicators in Severe OSA Patients Before and After nPAP in Followup**

|  | Sample1 | Sample2 | Sample3 | Sample4 | Sample5 | Sample6 | Sample7 | Sample8 | P value |
| --- | --- | --- | --- | --- | --- | --- | --- | --- | --- |
| **Pre-TCI** | 746 | 764 | 1644 | 1330 | 1386 | 1342 | 1353 | 1125 | **0.0078** |
| **Post-TCI** | 630 | 713 | 939 | 832 | 1015 | 835 | 766 | 545 |  |
| **Pre-Clone** | 5.57 | 13.77 | 2.56 | 2.49 | 1.64 | 4.9 | 2.42 | 5.58 | 0.3281 |
| **Post-Clone** | 3.79 | 3.45 | 9.41 | 1.05 | 1.84 | 3.64 | 3.17 | 4.83 |  |
| **Pre-Shannon** | 8.56 | 7.89 | 8.95 | 8.57 | 9.61 | 7.72 | 8.79 | 8.31 | **0.0469** |
| **Post-Shannon** | 8.97 | 9.38 | 8.81 | 9.08 | 9.65 | 7.97 | 9.02 | 8.31 |  |
| **Pre-D50** | 0.15 | 0.10 | 0.18 | 0.12 | 0.33 | 0.06 | 0.16 | 0.12 | **0.0078** |
| **Post-D50** | 0.20 | 0.30 | 0.22 | 0.19 | 0.34 | 0.08 | 0.20 | 0.14 |  |
| **Pre-NLR** | 1.68 | 1.91 | 1.8 | 2.89 | 1.37 | 1.57 | 2.15 | 3.32 | 0.8438 |
| **Post-NLR** | 1.56 | 1.33 | 1.83 | 1.81 | 1.88 | 1.92 | 2.02 | 3.57 |  |
| **Pre-PLR** | 131.47 | 101.02 | 186.92 | 110.91 | 77.93 | 50.18 | 152.63 | 125.93 | 0.8438 |
| **Post-PLR** | 151.6 | 100 | 119.17 | 97.1 | 60.13 | 58.63 | 173.38 | 156.36 |  |
| **Pre-CD4+/CD8+T** | 1.33 | 1.86 | 2.01 | 0.88 | 1.78 | 1.22 | 1.76 | 2.11 | 0.25 |
| **Post-CD4+/CD8+T** | 1.48 | 2.13 | 2.40 | 0.74 | 1.72 | 1.13 | 1.79 | 2.34 |  |
| **Pre-ESS** | 17 | 1 | 2 | 9 | 11 | 2 | 3 | 6 | 0.0938 |
| **Post-ESS** | 8 | 2 | 2 | 7 | 7 | 2 | 2 | 4 |  |
| Wilcoxon matched-pairs signed ranks test, significant level: P<0.05 | | | | | | | | | |
