# Supplementary figures and images for: T cell receptor repertoire as a novel indicator for identification and immune surveillance of patients with severe obstructive sleep apnea
Source: PeerJ. 2023 Apr 7;11:e15009. doi: 10.7717/peerj.15009 (PMC10084822; doi:10.7717/peerj.15009)

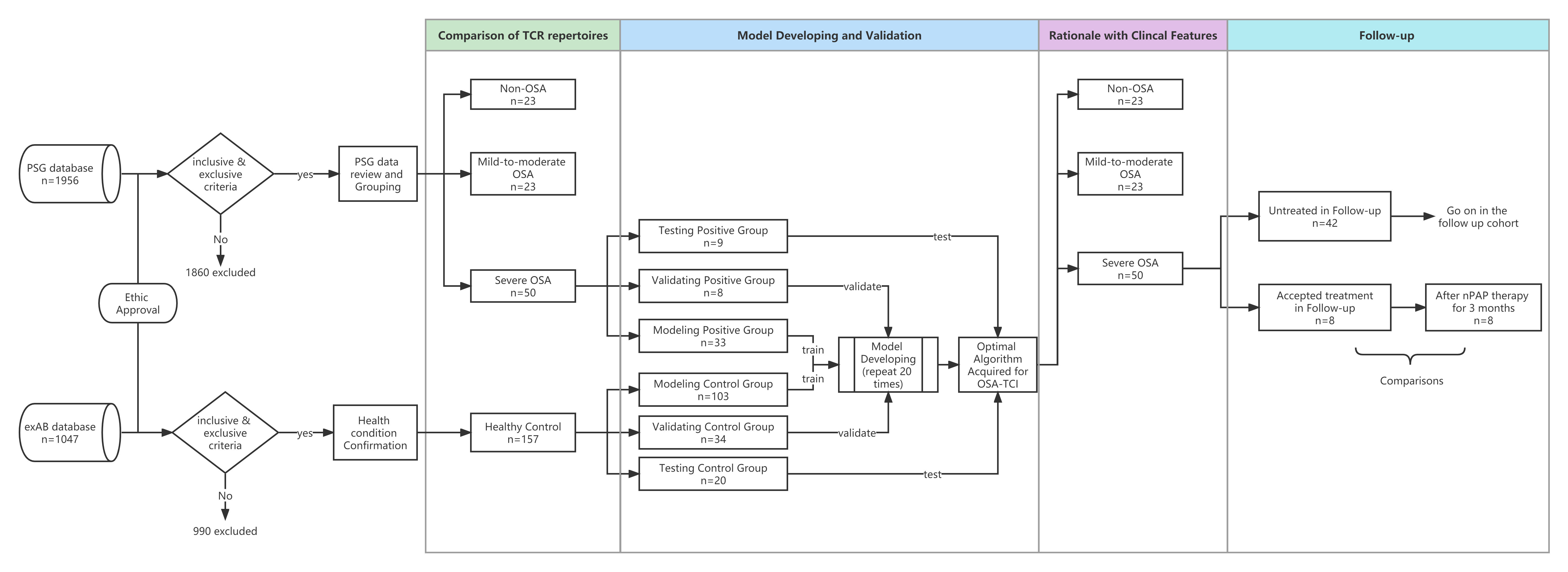

Supplement: Supplemental Information 8 [file peerj-11-15009-s008.png]

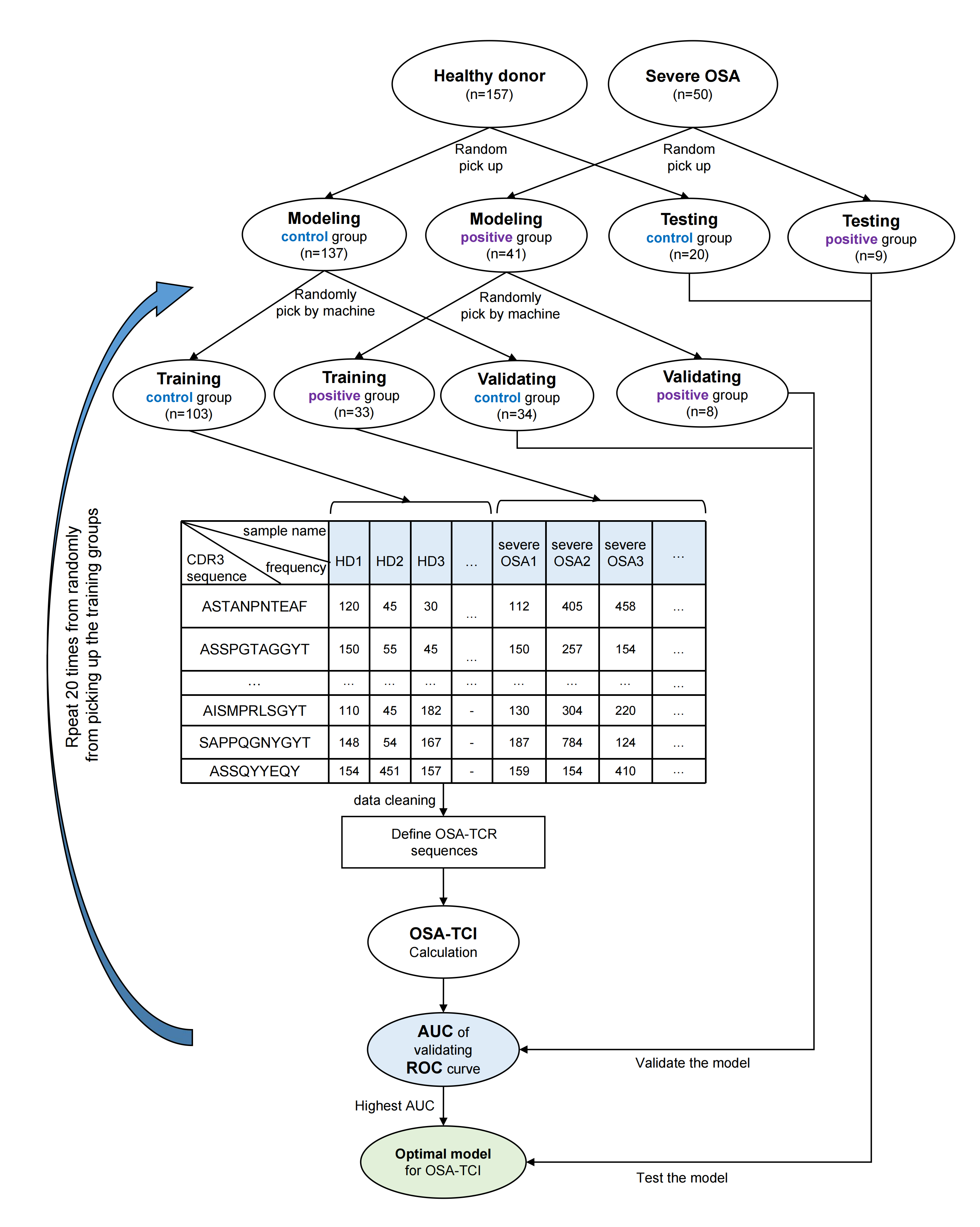

Supplement: Supplemental Information 9 [file peerj-11-15009-s009.png]

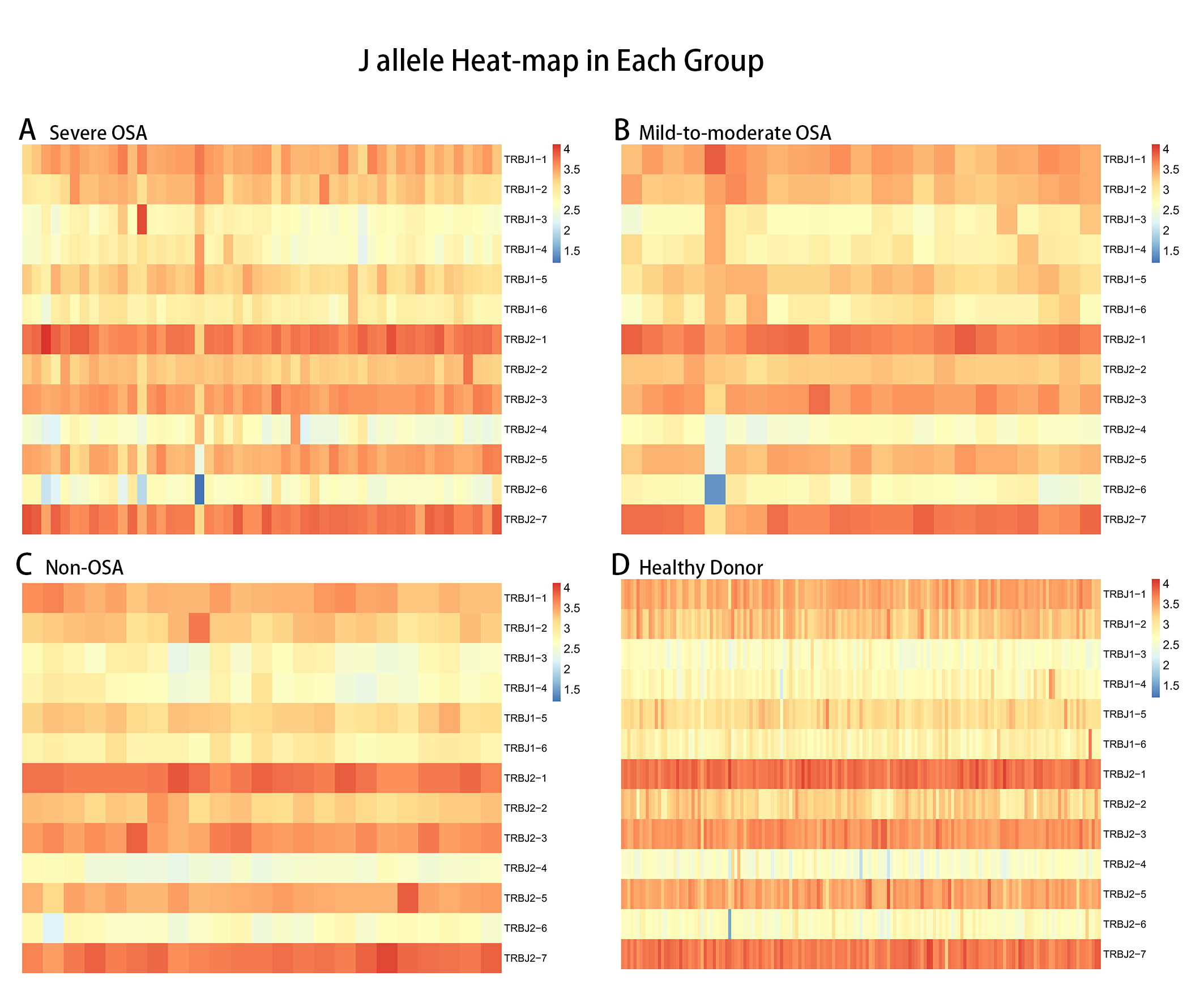

Supplement: Supplemental Information 10 [file peerj-11-15009-s010.png]

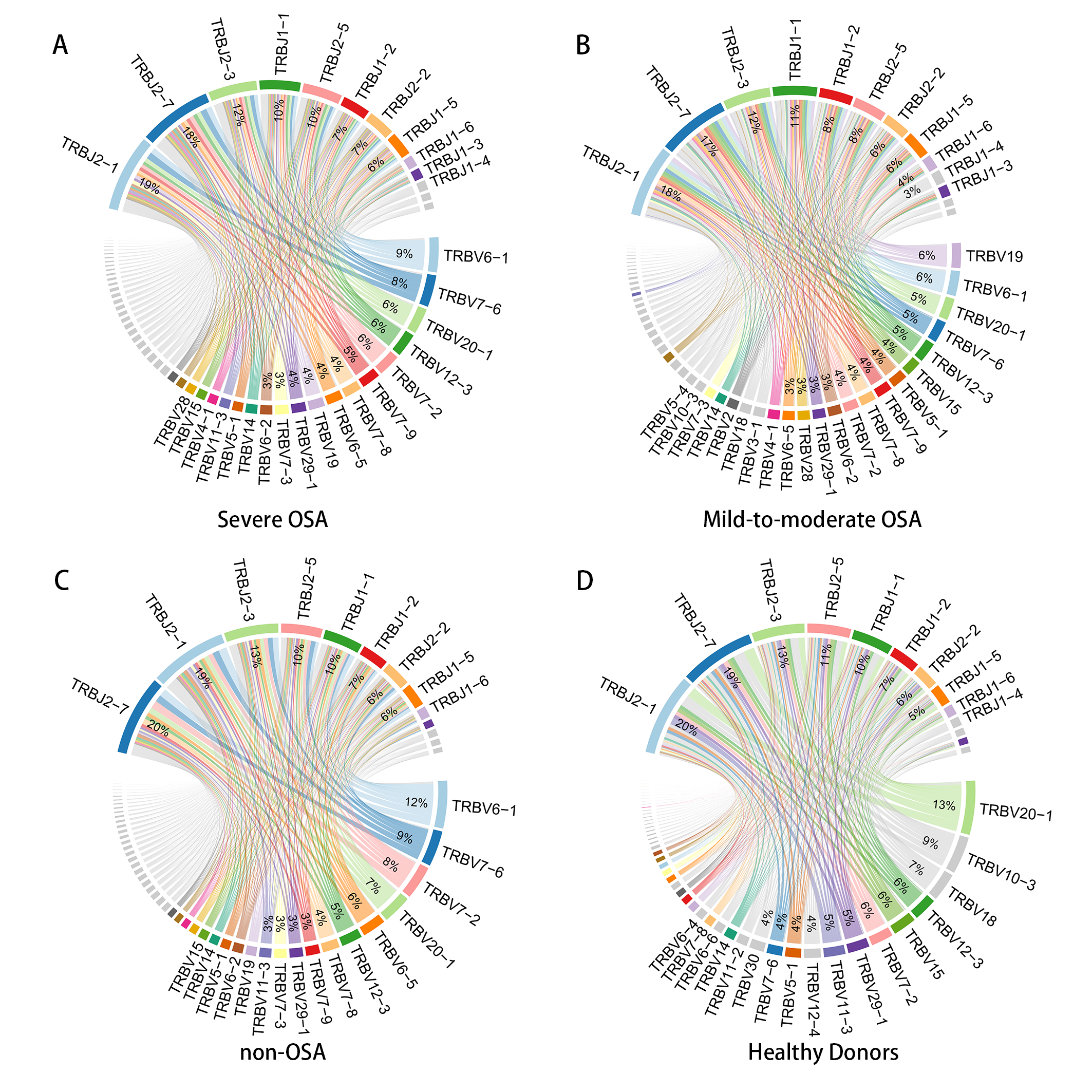

Supplement: Supplemental Information 11 [file peerj-11-15009-s011.png]

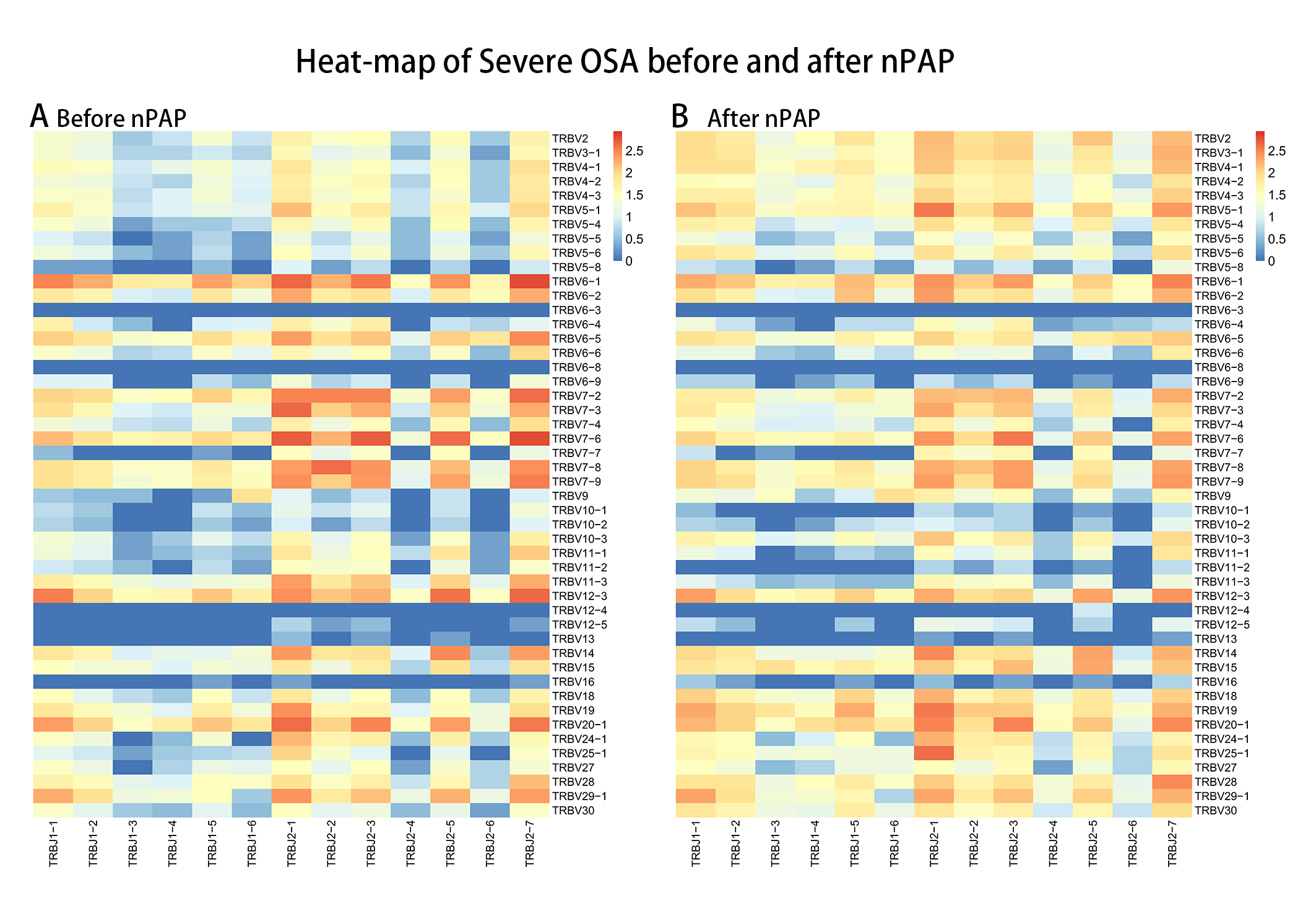

Supplement: Supplemental Information 12 [file peerj-11-15009-s012.png]

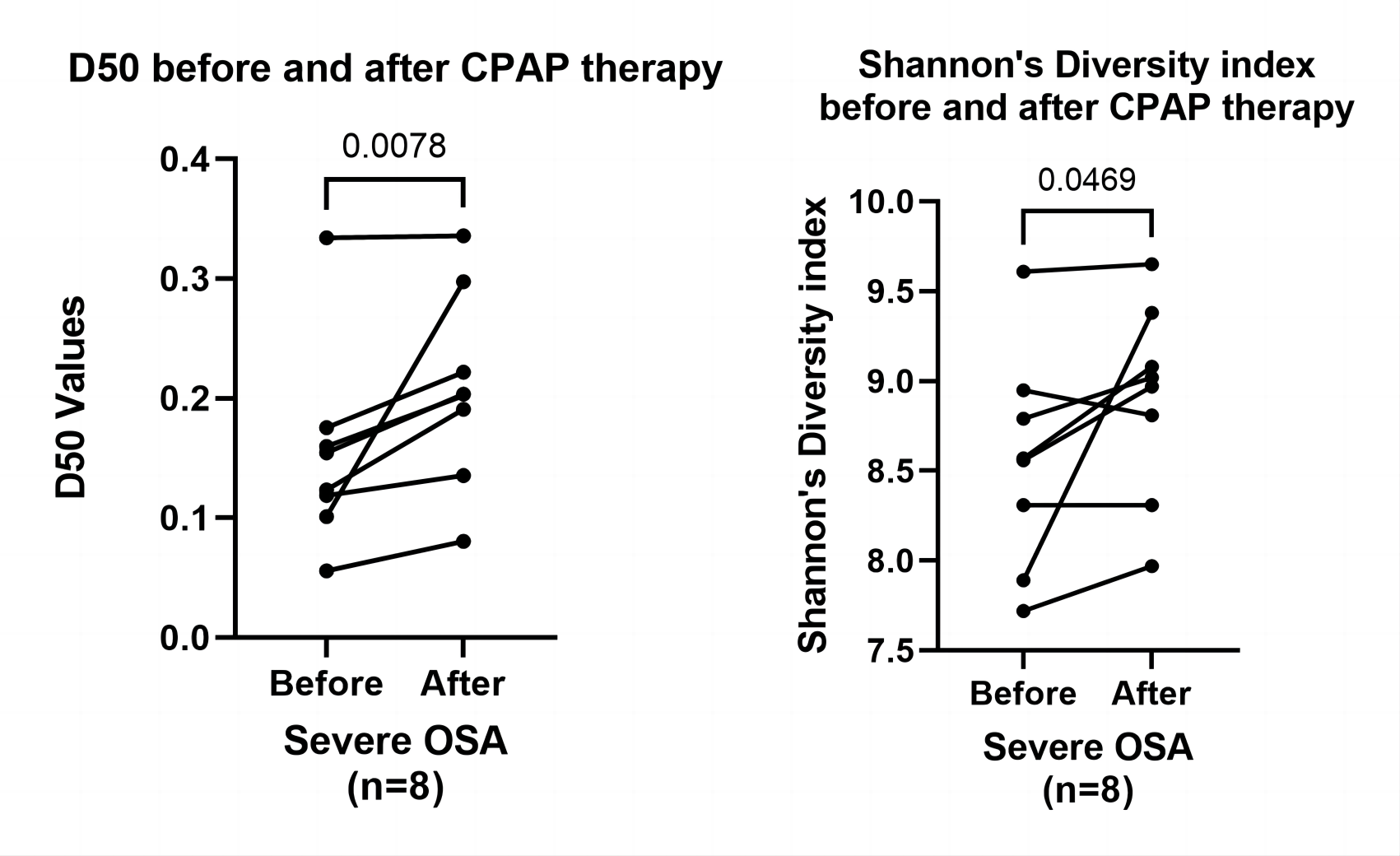

Supplement: Supplemental Information 13 — Paired comparison of D50 and Shannon’s Diversity Index before and after CPAP [file peerj-11-15009-s013.png]
